# The Hospitals and the Homes of England

**Published:** 1913-08-23

**Authors:** 


### THE HOSPITALS AND THE HOMES OF ENGLAND.

### Mr. Burns on Hospitals and Town Life.

The Right Hon. John Burns, President of the Local Government Board, in his address at the International Medical Congress on the 12th instant (the main institutional points in which were summarised in The Hospital of last week) on the Relationship between Medicine and Public Health, demonstrated clearly the enormous value of the voluntary hospital in the maintenance of the home life of the British people. On the increasing institutional treatment of disease Mr. Burns said:—

"I have already spoken of the increasing extent to which the sick are treated in institutions. Although at first sight the connection may not be obvious, I regard this as an essential branch of the subject of housing. If overcrowding is dangerous among those who are well, its danger is multiplied if the sick are commingled with the healthy; and it is almost certain that the universal excess of disease in town over country would have been much greater had it not been for the assistance given by the hospital to supplement domestic housing and nursing. It is not too much to claim for our hospitals that they have gone far towards removing the harmful effects of aggregation of populations in towns.

Think, for instance, not only of the relief to the patients themselves, but of the easing of the burden borne by the overworked family, which is involved in the simple statement that in the year 1911 in the whole of England and Wales 24 per cent., and in London 47 per cent., of the total deaths from cancer occurred in public institutions. But I am speaking to those who, with the philanthropists, have rendered this supremely beneficent work possible, and who have in very large measure carried it out; and I need, therefore, only add a few figures which will make still clearer the enormous extent to which the home has been relieved by the hospital. I am indebted for these figures to *Burdett's Hospital Annual.* In the United Kingdom there were in 1911, 49,854 beds in voluntary hospitals, or about 11 beds to 10,000 inhabitants, 80 per cent. of these being in daily occupation. The average stay for each patient was twenty-five days. These hospitals, including dispensaries, had in 1896 an income of £2,528,000, which in 1911 had increased to £4,185,000. The beds here enumerated do not include the much larger number in our workhouses and infirmaries, mental hospitals, isolation hospitals, etc."

